# Possible Role of High-Molecular-Weight Salivary Proteins in Astringency Development

**DOI:** 10.3390/foods13060862

**Published:** 2024-03-13

**Authors:** Elvira Manjón, Ignacio García-Estévez, María Teresa Escribano-Bailón

**Affiliations:** Department of Analytical Chemistry, Nutrition and Food Science, Universidad de Salamanca, E37007 Salamanca, Spain; elvira87@usal.es (E.M.); escriban@usal.es (M.T.E.-B.)

**Keywords:** α-amylase, mucin, human serum albumin, food flavan-3-ols, molecular interactions

## Abstract

Since the initial findings that food tannin/salivary protein interaction and subsequent precipitation is the main cause of the astringency development, numerous studies have concentrated on the supramolecular characterization of these bindings. Most of these works have focused on the low-molecular-weight salivary proteins, in particular proline-rich proteins, hardly considering the involvement of the high-molecular-weight salivary proteins (HMW_SPs_). Herein, different techniques such as fluorescence quenching, Isothermal Titration Calorimetry and HPLC-MS-DAD were employed to determine the occurrence of molecular interactions between three HMW_SPs_, namely, mucin, α-amylase and albumin, and a complex extract of tannins composed mainly of flavan-3-ols. The obtained results prove the capability of the three HMW_SPs_ to effectively interact with the flavan-3-ol extract, involving different forces and action mechanisms. Flavan-3-ols are capable of interacting with mucins by a mechanism that includes the formation of stable ground-state complexes that led to approximately 90% flavan-3-ol precipitation, while for albumin and α-amylase, the interaction model of a “sphere of action” was established, which represented only 20% flavan-3-ol precipitation. These data highlight the relevance of including HMW_SPs_ in astringency analyses, paying special heed to the role of mucins in the interaction and subsequent precipitation of dietary tannins.

## 1. Introduction

Astringency is commonly described as dryness, puckering, and a tightening sensation perceived in the mouth during the intake of certain foodstuffs rich in tannins [1]. The term tannin usually defines a particular group of polyphenols, like flavan-3-ols, that has the special ability to bind to proteins. Although various mechanisms have been described to elucidate the astringency sensation [2,3], the precipitation of salivary proteins in the oral cavity is the most widely accepted theory, generally attributed to the interaction with and subsequent precipitation of salivary proteins by tannins.

Human saliva, produced by the salivary glands, is rich in different types of peptides and proteins that are classified according to their structure and characteristics [4]. Regarding molecular weight, in human saliva, there are proteins with low molecular weight such as histatins, statherin, P-B peptide, cystatins or proline-rich proteins (PRPs) [5], but there are also other salivary proteins with higher molecular weight, such as α-amylases, mucins, lactoferrin, lysozyme, albumin or immunoglobulins, that represent an important fraction of the salivary proteome [6,7,8]. Huq and co-workers reported the concentrations (µg/mL) of salivary proteins and glycoproteins in whole human saliva based on the studies of other authors [7]. According to them, the most abundant high-molecular-weight salivary proteins are albumin (29–238 µg/mL), α-amylase (380–500 µg/mL), immunoglobulin A (19–439 µg/mL), mucin MG1 (80–500 µg/mL) and mucin MG2 (10–200 µg/mL); all of them were found in whole saliva in concentrations higher than that of PRPs (90–180 µg/mL) [7]. It has already been proved that low-molecular-weight salivary proteins, in particular PRPs, have a strong ability to interact with tannins, especially with flavan-3-ols, with interaction and consequent precipitation being the main mechanism responsible for the development of astringency [9]. However, the relationship of high-molecular-weight salivary proteins (HMW_SPs_) with astringency development has not been considered, despite their abundance in saliva.

Human salivary α-amylase accounts for about 20–30% (*w*/*w*) of salivary proteins, being one of the most plentiful proteins [8,10]. α-Amylase carries out a principal enzymatic function in starch digestion but is also able to bind with high affinity to a selected group of oral streptococci, a function that may contribute to bacterial clearance and nutrition [11]. It is a ~60 kDa protein produced by salivary glands, mainly by the parotid gland, that can be found in two forms in human saliva: a glycosylated isoform of ~62 kDa and a non-glycosylated form of ~56 kDa [12].

Albumins are a group of proteins found in the body fluids and tissues of animals and in some plant seeds. Human serum albumin is a carrier protein for a wide range of endogenous molecules, including hormones, fatty acids and metabolites, that is found in relevant amounts in saliva due to contamination by blood traces or by gingival crevicular fluids [4,13]. Human serum albumin, a single polypeptide chain of ~66 kDa with no carbohydrate content [14], is the major circulatory protein of well-known structure that is able to bind reversibly to a high variety of ligands [14,15].

Mucins, other major components of the salivary proteome (~16%), are highly glycosylated proteins consisting of ~80% carbohydrates [16]. There are two main mucins secreted in the oral cavity: mucin 5 and mucin 7. Mucin 5, also known as high-molecular-weight salivary mucin or MG1, because it has a size of >1 MDa, forms a viscoelastic network that is important in the oral cavity for hydration, lubrication, pathogen exclusion and resistance to proteolytic digestion. For its part, mucin 7, also known as low-molecular-weight human salivary mucin or MG2, is an antimicrobial protein of ~200 kDa that has a role in aiding the clearance of bacteria from the oral cavity and also helps in mastication, speech and swallowing [17,18].

Since these three HMW_SPs_ are implicated in roles in which they can bind to other proteins, it is possible that some of them could be related to astringency development by binding to tannins, thus avoiding or modulating the PRP/tannin interaction. Scarce studies have deeply examined the interactions between tannins and different HMW_SPs_ in order to achieve the physicochemical characterization of the resulting interactions [19,20,21]. Moreover, these studies employed different tannins, analytical techniques or conditions, making it difficult to compare the obtained results. To date, no study has been carried out in which the main HMW_SPs_, namely, mucin, α-amylase and albumin, have been analyzed under the same conditions. Likewise, the nature of the resulting interaction complexes and the molecular forces implicated in their formation have not been elucidated.

Herein, to unravel the molecular mechanisms underpinning the complex sensation of astringency, the occurrence of interactions between the major HMW_SPs_ (α-amylase, mucin and albumin) and a complex flavan-3-ol extract, composed of monomers and polymers with and without galloyl residues, is determined and fully characterized by combining techniques such as fluorescence quenching, Isothermal Titration Calorimetry (ITC) and HPLC-DAD-MS^2^.

## 2. Materials and Methods

### 2.1. Seed Flavan-3-ol Extract

Seeds from ripe *Vitis vinifera* L. cv Tempranillo grapes were manually collected, lyophilized and ground. The resulting powder was extracted three times with ethanol/water (75:25, *v*/*v*) to obtain a seed extract that was purified using a C18 solid-phase cartridge, and elution was performed with 20% ethanol to obtain a flavan-3-ol extract composed mainly of monomers and oligomers, similar to those used in other reported astringency assays. The composition of the resulting extract was determined by HPLC–DAD–MS^2^, following the procedure described by García-Estévez and co-workers [22]. In total, 53 compounds were identified and quantified: 2 monomers, 9 dimers, 17 trimers, 17 tetramers and 8 pentamers, from which 4 dimers, 6 trimers and 6 tetramers were galloylated. As can be observed in Table S1 of the Supplementary Materials, the flavan-3-ol extract was composed mainly of oligomers. The content of non-galloylated flavan-3-ols was ∼88%, while the galloylated ones represented ∼12%. The average molecular weight and the medium degree of polymerization were ∼621 g/mol and ∼2.1, respectively.

### 2.2. High-Molecular-Weight Salivary Proteins (HMW_SPs_)

The high-molecular-weight salivary proteins (HMW_SPs_) employed here, namely, bovine submaxillary mucin (type I-S), α-amylase from human saliva (type XIII-A) and albumin from human serum were acquired from Sigma-Aldrich (St. Louis, MO, USA) with a purity of ≥95%. The molecular weight (MW), total amino acid content, and percentages of hydrophobic, polar, acid, basic and aromatic residues of these HMW_SPs_, according to the UniProt database, are shown in Table S2 of the Supplementary Materials.

### 2.3. Fluorescence Quenching

The occurrence of interactions between each HMW_SP_ and the flavan-3-ol extract was analyzed by using fluorescence quenching measurements. The intrinsic fluorescence of proteins is usually due to the presence of aromatic amino acids such as phenylalanine, tyrosine and tryptophan, the latter one being the most strongly related to this process [23]. The intrinsic fluorescence of samples was determined by using a Perkin-Elmer LS 55 fluorescence spectrophotometer (Waltham, MA, USA) in 1 cm quartz cuvettes at a controlled temperature (283 K). The excitation wavelength (λ_ex_) for all HMW_SPs_ was set at 290 nm, and the emission spectra were recorded from 300 nm to 500 nm. For all experiments, HMW_SPs_ and flavan-3-ol stock solutions were dissolved in distilled water. Different HMW_SP_ concentrations were assayed; 3 µM of α-amylase, 3 µM of albumin and 0.1 µM of mucin were the concentrations employed for the experiments, since they provided a sufficient signal without reaching saturation. The preparation of the interaction samples was performed in 0.5 mL microtubes, where increasing volumes of the flavan-3-ol stock solution (400 µM) were added to each HMW_SP_ solution, reaching the following final concentrations of flavan-3-ols: (i) 0–255 µM for the interactions with α-amylase, (ii) 0–150 µM for the interactions with albumin and (iii) 0–60 µM for the interactions with mucin. Since flavan-3-ols absorb energy at the established emission wavelength, a blank assay was made for each flavan-3-ol concentration, wherein the HMW_SP_ solution was replaced by distilled water. Right after mixing, the microtubes were shaken and left at room temperature for a duration of 10 min to allow the interactions to take place. Then, the emission spectra of each interaction and its corresponding blank samples were measured in the fluorimeter cell. All experiments were performed in triplicate.

### 2.4. Isothermal Titration Calorimetry (ITC)

With the aim of obtaining the thermodynamics parameters associated to each HMW_SP_/flavan-3-ol interaction, a MicroCal PEAQ-ITC system (Malvern, UK) was used to measure the heat generated or absorbed upon the corresponding interactions between each HMW_SP_ and the flavan-3-ol extract. The concentrations of each protein and the flavan-3-ols for each interaction were selected to record a sufficient energy signal and to achieve saturation of the interaction process. In brief, 200 μM of the flavan-3-ol solution was used to titrate α-amylase (5 μM); 500 μM of the flavan-3-ol solution was used to titrate albumin (5 μM); and 700 μM of the flavan-3-ol solution was used to titrate mucin (0.4 μM). All solutions were prepared in ultrapure water. In all experiments, the flavan-3-ol solution was loaded into the injection syringe, and each HMW_SP_ solution was placed into the 0.2 mL sample cell of the calorimeter. Blank experiments (flavan-3-ols/water and water/HMW_SP_) were also conducted. The syringe solution was titrated into the sample cell at a constant temperature of 283 K as a sequence of 19 injections of 2 μL aliquots with one initial delay (time for equilibration) of 300 s. The content of the sample cell was stirred throughout the experiment at 750 rpm.

The thermodynamic parameters, such as the binding apparent constant (*K*), change in Gibbs free energy (Δ*G*), enthalpy (Δ*H*) and entropy (−*T*Δ*S*), were determined by using the software AFFINIMETER (https://www.affinimeter.com/site/) (Software for Science Developments, Santiago de Compostela, Spain). An independent sites model with two sets of sites (two different types of sites for the interaction) was employed for the fitting, in which blank experiments were subtracted from the corresponding interactions. All experiments were performed in triplicate.

### 2.5. HPLC-DAD-MS^2^ Analysis

To deepen the interaction studies, an analysis of the solubility of the corresponding complexes was carried out by HPLC-DAD-MS^2^. For this, the molar ratio of each biomolecule (dissolved in ultrapure water) was selected according to the quenching results; specifically, molar ratios were selected at which the initial HMW_SP_ fluorescence decreased to 50% after interaction (1:55 for α-amylase/flavan-3-ols, 1:20 for albumin/flavan-3-ols and 1:250 for mucin/flavan-3-ols). For each interaction assay, a flavan-3-ol control was also prepared by mixing the flavan-3-ol solution with the corresponding volume of ultrapure water. Interactions took place at room temperature for 10 min. Afterwards, the samples were centrifuged (13,709× *g*, 5 min) and the supernatants were immediately analyzed by HPLC-DAD-MS^2^ following the procedure described by García-Estévez and co-workers [22]. All interaction assays were performed in triplicate.

### 2.6. Statistical Analysis

For the statistical analysis, IBM-SPSS Statistics 26 software was employed. Differences were assessed through a one-way analysis of variance (ANOVA) followed by a post hoc Tukey-B test, with significance set at *p* < 0.05. The flavan-3-ol composition changes determined by HPLC-DAD-MS^2^ were evaluated using Student’s t-test to compare the control and the interaction samples.

## 3. Results and Discussion

As mentioned in the Introduction (Section 1), the ability of HMW_SPs_ to interact with procyanidins has been already pointed out by different authors, indicating a possible involvement in the sensation of astringency. These assays are usually performed with model HMW_SPs_, which are usually not obtained from saliva and, in some cases, are not obtained from humans; for example, Brandão and coworkers (2017) and Gombau and coworkers (2019) employed mucin type III from porcine stomach for their molecular assays [19,24]; Ferrer-Gallego and coworkers (2012) and Soares and coworkers (2009) used α-amylase from porcine pancreas [20,25]; while De Freitas and Mateus (2001) separated human saliva into two fractions, one rich in PRPs and another in α-amylase, but also employed albumin from bovine serum [21]. Here, the commercial HMW_SPs_ employed are structurally similar to the actual HMW_SPs_, and their interactions with an extract of oligomeric procyanidins with different mean degrees of polymerization were observed and characterized by different techniques.

### 3.1. Fluorescence Quenching Analysis

**Figure 1:**
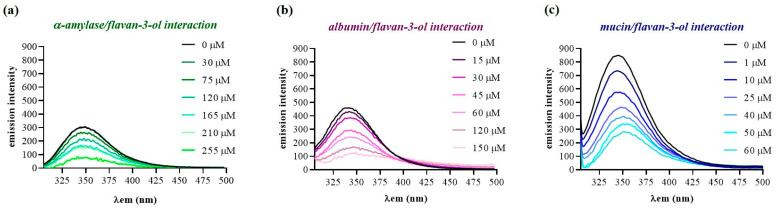
Fluorescence emission spectra (at λ_ex_ 290 nm) of each HMW_SP_ in the presence of increasing concentrations of the seed flavan-3-ol extract: (**a**) α-amylase (3 µM); (**b**) albumin (3 µM); (**c**) mucin (0.1 µM). Each curve represents a triplicate assay after correction for flavan-3-ol fluorescence.

The occurrence of molecular interactions between each HMW_SP_ and the flavan-3-ol extract was determined by fluorescence quenching. This technique is a rapid and useful method for establishing bindings and conformational changes in proteins, which has been previously used for determining the decrease in the quantum yield of fluorescence from proteins containing tryptophan residues (fluorophores) after molecular interactions with different phenolic compounds [10]. All HMW_SPs_ employed in this work contain tryptophan amino acid units in their sequence and, thus, they are good candidates to be studied in quenching assays. Figure 1 shows the fluorescence emission spectra (300–500 nm) obtained for α-amylase (Figure 1a), albumin (Figure 1b) and mucin (Figure 1c) after adding increasing concentrations of the flavan-3-ol extract. These spectra were obtained by subtracting the spectra of each respective flavan-3-ol solution, as also performed by other authors [26]. As can be seen in Figure 1, after adding increasing contents of flavan-3-ols to each HMW_SP_ solution, there was a gradual decrease in the protein fluorescence intensity, which confirmed that flavan-3-ols can bind to all of the HMW_SPs_ studied.

**Figure 2:**
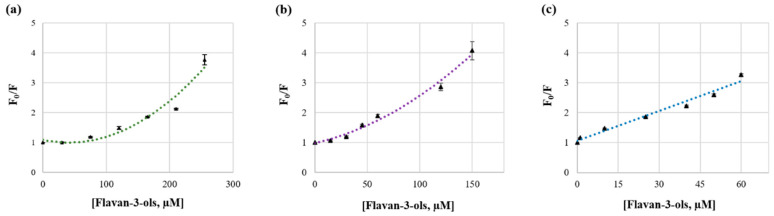
Stern–Volmer plots describing tryptophan quenching of HMW_SPs_ by increasing concentrations of seed flavan-3-ols: (**a**) amylase (3 µM); (**b**) albumin (3 µM); (**c**) mucin (0.1 µM). The λ_em_ maximum was recorded at ~350 nm.

Fluorescence quenching is typically described by the Stern–Volmer equation (Equation (1)):

F_0_/F = 1 + *k_q_ τ*_0_[Q] = 1 + *K*_SV_[Q] (1)

where F_0_ and F are the fluorescence intensities before and after the addition of the quencher, respectively; *k_q_* is the bimolecular quenching constant; *τ*_0_ is the lifetime of the fluorophore in the absence of quencher; [Q] is the concentration of the quencher and *K*_SV_ is the Stern–Volmer quenching constant [27]. This equation is usually applied to determine the affinity constant, *K*_SV_, by linear regression of a plot of F_0_/F against [Q]. The resulting plots for each HMW_SP_ at the λ_em_ maximum (~350 nm) are shown in Figure 2.

As can be seen, the Stern–Volmer plot obtained for mucin/flavan-3-ol interaction was linear (Figure 2c), which is generally indicative of a single type of fluorophore in a protein, displaying all units having equal accessibility to the quencher. This also means that only one mechanism of quenching occurs (dynamic or static). It is worth emphasizing that both static and dynamic quenching require molecular contact between the fluorophore and the quencher, which supports the occurrence of interactions. To study this interaction more deeply, the bimolecular quenching constant, *k_q_*, was calculated by dividing the value of *K_SV_* by *τ*_0_ (3.68 × 10^−9^ s) according to Equation (1). The obtained value for *k_q_,* 9.06 × 10^12^ M^−1^ s^−1^ (Table S3 of the Supplementary Materials), was higher than the maximum value possible for diffusion-limited quenching or a dynamic mechanism (10^10^ M^−1^ s^−1^), which points out that flavan-3-ols interact with mucin by a static mechanism, involving the formation of a stable ground-state complex between the two biomolecules [27,28]. This result is in agreement with the results published by Brandão and coworkers (2017), who found linear Stern–Volmer plots describing the quenching of porcine mucin by increasing concentrations of different fractions of oligomeric procyanidins [19].

On the contrary, the resulting Stern–Volmer plots for the interactions with α-amylase (Figure 2a) and with albumin (Figure 2b) show a concave curvature at high flavan-3-ol concentrations, distinctive of upward-curving Stern–Volmer plots. These plots may suggest (i) that the fluorophore is being quenched by two mechanisms simultaneously—collisions (dynamic mechanism) and complex formation (static mechanism)—with the same quencher or (ii) the existence of a “sphere of action”. This second model (ii) assumes the existence of a sphere around the fluorophore within which quenching occurs due to the quencher being adjacent to the fluorophore at the moment of excitation, without the formation of a ground-state complex [27]. In these cases, the fluorescence quenching obeys the modified form of the Stern–Volmer equation described by the “sphere of action model” [19,29] (Equation (2)):

ln (F_0_/F) = f [Q] (2)

where F_0_ and F are the fluorescence intensities before and after the addition of the quencher, respectively; [Q] is the concentration of the quencher. The resulting modified Stern–Volmer plots for α-amylase and albumin interactions (Figure S1 of the Supplementary Materials) allow us to calculate the apparent static quenching constant (*K_app_*) and the apparent bimolecular quenching constant (*K_q_^app^*) from the ratio between *K_app_* and *τ_0_*. To calculate *K_q_^app^*, according to previous studies, the average lifetimes selected were 2.97 ns for α-amylase [30] and 6.38 ns for human serum albumin [31]. The obtained values for *K_q_^app^* (Table S3 of the Supplementary Materials) were both higher than 10^10^ M^−1^ s^−1^, which confirmed the occurrence of a purely static mechanism.

### 3.2. Isothermal Titration Calorimetry Assays

ITC experiments have already been employed to successfully characterize the bindings underlying the astringency related to the interaction between low-molecular-weight salivary proteins and different phenolic compounds [32,33]. This effective methodology was employed herein to thoroughly study the interactions found previously by fluorescence quenching, by analyzing the thermodynamic parameters associated to each interaction process. Table 1 compiles the thermodynamic parameters obtained from the ITC data, namely, the number of binding sites per molecule (*n*), the binding apparent constant (*K*), and the changes in enthalpy (Δ*H*) and Gibbs free energy (Δ*G*) calculated from the binding constant (Δ*G* = −*RT* ln *K*, where *R* is the gas constant and *T* is the absolute temperature in Kelvin) and the entropic component obtained from the second law of thermodynamics (Δ*G* = Δ*H* − *T*Δ*S*).

The occurrence of spontaneous interactions between flavan-3-ols and the three HMW_SPs_ was revealed, since all of the Δ*G* values were negative (Table 1). Moreover, the more negative the Δ*G* value, the more spontaneous the process is, which points out that flavan-3-ols showed greater affinity for albumin than for α-amylase and, in turn, greater affinity for these two proteins than for mucin, which was also proved by the *K* values obtained.

However, despite the determined *K* values being the lowest for the mucin/flavan-3-ol system, it is worth noting that a significantly higher number of binding sites (*n*) was detected for this system than for the albumin or α-amylase interactions (Table 1). This could be due to the larger size of the mucin compared to the other two HMW_SPs_ assayed or could be related to the fact that for the formation of a stable ground-state complex, a greater number of binding sites is necessary. This second hypothesis would be in agreement with the quenching results and would suggest that, even with lesser affinity, flavan-3-ols are capable of binding to mucin in different and numerous positions, which could lead to the formation of stable complexes with a high content of flavan-3-ols attached. On the other hand, the driving forces that govern these interactions were deduced from the Δ*H* and −*T*Δ*S* values. Hydrophobic interactions are the main forces when the process is entropy driven (Δ*H* > 0 and −*T*Δ*S* < 0), whereas enthalpy-driven interactions (Δ*H* < 0, −*T*Δ*S* > 0) are related to exothermic H-bonds [34]. According to our results, both H-bonds and hydrophobic forces are involved in the mucin/flavan-3-ol and albumin/flavan-3-ol systems, since the Δ*H* and −*T*Δ*S* values were both negative for the two sets (Table 1); this is in concordance with the information described for the interactions between flavan-3-ols and low-molecular-weight salivary proteins [32]. However, it should be pointed out that in the α-amylase/flavan-3-ol interaction, only hydrophobic forces were detected (Δ*H* > 0 and −*T*Δ*S* < 0). It has been described that the polyphenol/salivary protein interactions occur firstly through hydrophobic stacking between the galloyl ring of the polyphenol and the pyrrolidine ring face of proline amino acid. Then, secondary H-bonds help to stabilize the complexes [9]. Herein, the amount of proline residues in the α-amylase amino acid sequence was similar to that found in the albumin sequence, namely, ~4.3% of the protein residues are prolines in both HMW_SPs_, which could suggest that the proline content is not the cause of the different forces implicated. On the contrary, the aromatic residue content, which could also be implicated in the hydrophobic bonds, does represent a different fraction in each HMW_SP_ (Table S2 of the Supplementary Materials). α-Amylase contains 12.9% aromatic residues, of which 26 are phenylalanine amino acids, 21 are tyrosine residues and 17 are tryptophan amino acids, while human serum albumin is composed of 31 phenylalanine amino acids and 18 tyrosine residues but only 1 tryptophan amino acid; i.e., albumin contains 8.5% aromatic residues (UniProt database) [10,35]. These data might suggest that the hydrophobic forces found in the α-amylase/flavan-3-ol system could be related with its high content of tryptophan amino acids.

### 3.3. HPLC-DAD-MS^2^ Assays

An evaluation of HMW_SP_/flavan-3-ol interactions was also performed by HPLC-DAD-MS^2^ in order to look deeper into the specific families of flavan-3-ols that are able to interact with each HMW_SP_. The flavan-3-ol extract employed was composed of 53 procyanidins, 16 of them galloylated. Within the non-galloylated compounds, five different families were found: monomers (2), dimers (5), trimers (11), tetramers (11) and pentamers (8), representing ~88% of the extract. Also, three families of galloylated flavan-3-ols were determined in the extract (~12% of the extract): dimers (4), trimers (6) and tetramers (6). This flavan-3-ol extract showed a medium degree of polymerization of 2.09, and it was similar in composition (Table S1 of the Supplementary Materials) to the flavan-3-ol extracts employed in other astringency studies [36,37].

**Figure 3:**
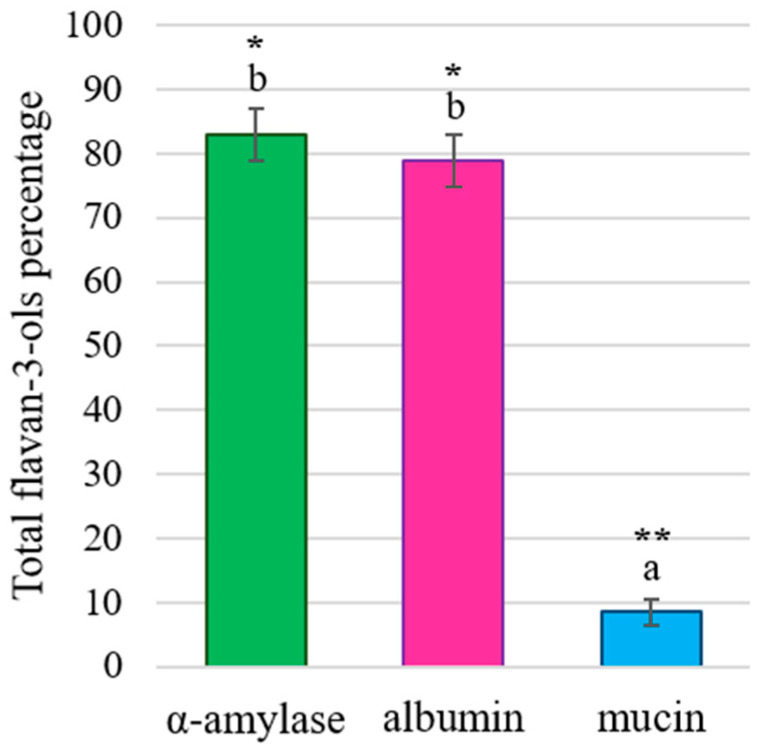
Percentages (%) of the total flavan-3-ols detected in solution, taking the original extract (control) as a reference, after interaction with α-amylase (green), albumin (pink) or mucin (blue). Different letters indicate statistically significant differences (*p* < 0.05) among samples. Significant differences between the control and the interaction samples are indicated with * (*p* < 0.01) or ** (*p* < 0.001).

As can be observed in Figure 3, all HMW_SPs_ were able to bind and to significantly precipitate flavan-3-ols, since the content of soluble flavan-3-ols detected by HPLC-DAD-MS^2^ was lower than the content of the control sample. However, it is worth noting that both α-amylase and albumin precipitated ca. 20% of the total flavan-3-ols, without significant differences between them, while mucin showed a much more relevant effect, being capable of precipitating up to 90% of the analyzed flavan-3-ols.

These results suggest that the ground-state complexes formed between mucin and flavan-3-ols are not actually soluble and finally precipitate, dragging, as a consequence, a considerable amount of flavan-3-ols with them. Proteins that are rich in proline generally exhibit a stronger interaction with tannins. Indeed, the presence of proline amino acids in proteins leads to some structural constraints, since it is the only residue where the side chain connects to the protein backbone twice, forming a pyrrolidine side chain, which gives exceptional conformation rigidity [38]. Mucins generally include a long central domain rich in proline, threonine and serine (PTS domain), tightly attached to anionic and hydrophilic carbohydrate chains. The glycosylation level of the PTS domain reaches 70−85% of the total molecular weight, where sialic acid accounts for as much as 30% [39]. These high proline and carbohydrate contents cause mucin to be considered more rigid than the other assayed HMW_SPs_, which could boost the insolubility of the resulting aggregates with flavan-3-ols, helping their precipitation.

**Figure 4:**
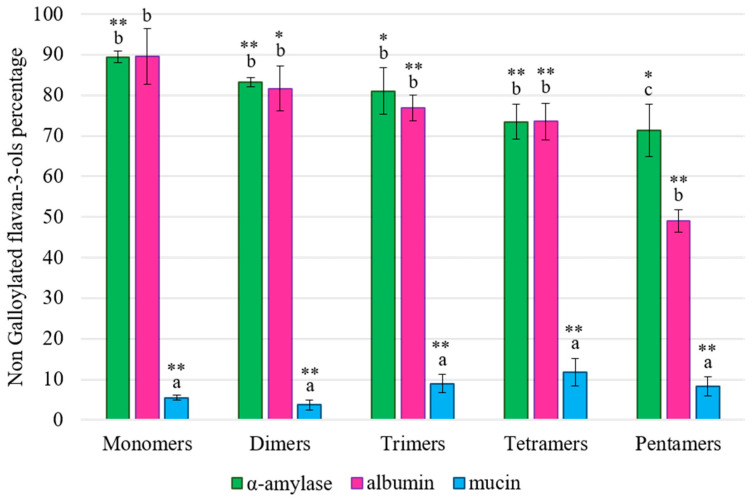
Percentages (%) of non-galloylated flavan-3-ols detected in solution, taking the original extract (control or 100%) as a reference, after interactions with α-amylase (in green), albumin (in pink) or mucin (in blue). Different letters indicate statistically significant differences (*p* < 0.05) among samples. Significant differences between the control and the interaction samples are indicated with * (*p* < 0.01) or ** (*p* < 0.001).

Regarding the different families of flavan-3-ols, it is accepted that salivary proteins, in particular PRPs, show stronger interaction and precipitation of specially galloylated flavan-3-ols with a high degree of polymerization [40,41]. In this work, a quantification of the different families found in the employed extract was performed. Regarding non-galloylated compounds (Figure 4), it was found that, again, α-amylase and albumin showed similar behavior with regards to the substrate preference, without significant differences in their effect on monomer, dimer, trimer or tetramer levels. In both cases, it can be observed that the higher the degree of polymerization is, the more flavan-3-ol/HMW_SP_ interactions and precipitation occur, with pentamers being the only family for which significant differences in their amount were observed when the interactions with α-amylase and albumin were compared. However, since the amount of pentamers in the total extract was low (see Table S1 of the Supplementary Materials), this difference between α-amylase and albumin was not expected to translate into a notable effect on the total content of flavan-3-ols. Nevertheless, it is possible that in other extracts or food that would be richer in pentamers, albumin would show a significantly greater effect than α-amylase on the precipitation levels. It is important to mention that both the ITC (Table 1) and quenching (Table S3 of the Supplementary Materials) data showed higher affinity constants for albumin than for α-amylase.

As aforementioned, the effect of the procyanidin polymerization degree on interactions with proteins has been already reported. For example, De Freitas and Mateus (2001) established that different salivary proteins showed higher interactions with procyanidin trimer C1 than with procyanidin dimers or monomers [21], a trend that was also observed in our results in the case of α-amylase and albumin but, curiously, not for mucin.

With respect to the effect of mucin on the precipitation of non-galloylated flavan-3-ols (Figure 4), a clear relationship between the flavan-3-ols’ polymerization degree and their binding to the protein was not found, since the compounds that precipitated the most were mainly dimers, suggesting that this HMW_SP_ does show a possible substrate preference compared to the other salivary proteins assayed. Likewise, it is important to highlight that the differences between mucin and control samples were highly significant (*p* < 0.001) in all families assayed, supporting the notable effect of mucin on flavan-3-ol precipitation.

**Figure 5:**
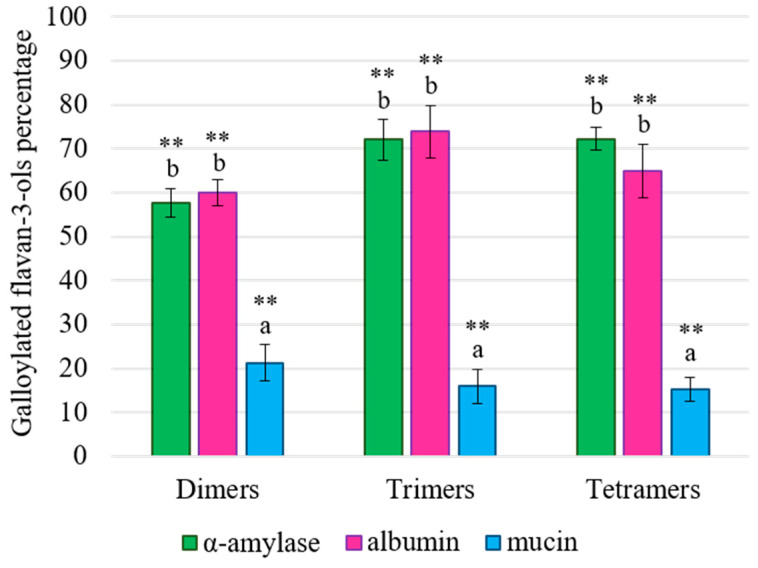
Percentages (%) of galloylated flavan-3-ols detected in solution, taking the original extract (control or 100%) as a reference, after interactions with α-amylase (in green), albumin (in pink) or mucin (in blue). Different letters indicate statistically significant differences (*p* < 0.05) among samples. Significant differences between the control and the interaction samples are indicated with ** (*p* < 0.001).

The effect on the galloylated flavan-3-ols was also determined (Figure 5). As can be seen from the figure, again, there were no significant differences between the results obtained for the interactions involving α-amylase and albumin, although it is worth highlighting that the differences with the control sample were more important for galloylated compounds than for non-galloylated ones. For galloylated compounds, ca. 70% remained in solution after the interactions (Figure 5), while for the non-galloylated ones, this level was only reached for the tetramer and pentamer families (Figure 4).

Moreover, it is remarkable that the relationship between the polymerization degree and higher precipitation that was found for the non-galloylated flavan-3-ols (Figure 4) was not detected for the galloylated ones (Figure 5). This fact could point out that the presence of the galloyl group is key for the interaction with α-amylase and albumin, and it would be in concordance with the data published by other authors. In fact, De Freitas and Mateus also observed an increase in the interaction between flavanols and proteins such as PRPs, α-amylase or bovine serum albumin (BSA) due to galloylation, since the procyanidin dimer B2-3′-*O*-gallate showed greater interaction than its counterpart, procyanidin dimer B2 [21,42].

Altogether, these data suggest that the effect found on the total flavan-3-ol content for α-amylase and albumin could be due to the fact that both HMW_SPs_ bound mainly to galloylated flavan-3-ols, showing a sort of substrate specificity for the galloylated compounds. On the contrary, mucin was capable to bind to galloylated flavan-3-ols regardless of their degree of polymerization (Figure 5), which pointed out that this HMW_SP_ did not show any special affinity for a single galloylated family, but, rather, it was capable of interacting with different flavan-3-ols in a stronger way, probably leading to large complexes that, in turn, precipitated with several flavan-3-ols attached.

## 4. Conclusions

In this work, it was proved that aside from low-molecular-weight salivary proteins, other proteins that can be found in human saliva can also interact with dietary phenolic compounds, in particular with different families of flavan-3-ols; consequently, they could also participate in the development of the sensation of astringency. For the first time, the molecular interactions that take place between HMW_SPs_ and a complex extract of flavan-3-ols were characterized under the same conditions, through different techniques such as fluorescence quenching, ITC and HPLC-MS-DAD^2^, which allowed us to perform more accurate comparisons between the HMW_SPs’_ behaviors. The overall results confirmed that flavan-3-ols are capable of interacting with the three HMW_SPs_ assayed, by means of different mechanisms and binding forces, with special attention to the role of mucins in the interaction with and precipitation of phenolic compounds. These interactions may lead to a significant impact on the physiological functions of the HMW_SPs_, which would be interesting to study due to their enzymatic or carrier functions, opening the door to future research. In recent years, substantial progress has been made in expanding our understanding on the molecular basis of astringency, and the results of this work confirm that the development of astringency involves not only several mechanisms but also proteins other than PRPs, which highlights the need to carry out more reliable astringency studies that include HMW_SPs_.

## Figures and Tables

**Table 1:** Thermodynamic parameters for the α-amylase/flavan-3-ol, albumin/flavan-3-ol and mucin/flavan-3-ol interactions. Different letters indicate statistical differences (*p* < 0.05) among interaction samples.

	α-amylase/flavan-3-ols	albumin/flavan-3-ols	mucin/flavan-3-ols
Δ*G_Total_* (cal mol^−1^)	(−2.818 ± 0.001) × 10^4^ b	(−2.950 ± 0.001) × 10^4^ a	(−2.802 ± 0.001) × 10^4^ c
Set 1			
n_1_	5.5 ± 0.2 a	2.8 ± 0.3 a	81.2 ± 0.8 b
*K*_1_ (M^−1^)	(1.25 ± 0.08) × 10^11^ b	(2.06 ± 0.05) × 10^11^ c	(4.1 ± 0.4) × 10^10^ a
Δ*H*_1_ (cal mol^−1^)	(1.1 ± 0.1) × 10^3^ c	(−2.5 ± 0.3) × 10^3^ b	(−7.1 ± 0.8) × 10^3^ a
*−T*Δ*S*_1_ (cal mol^−1^)	(−1.62 ± 0.01) × 10^4^ a	(−1.30 ± 0.03) × 10^4^ b	(−7.4 ± 0.9) × 10^3^ c
Set 2			
n_2_	5.3 ± 0.6 a	13.5 ± 0.2 b	187.6 ± 0.2 c
*K*_2_ (M^−1^)	(3.7 ± 0.4) × 10^9^ a	(2.09 ± 0.08) × 10^10^ c	(8.8 ± 0.4) × 10^9^ b
Δ*H*_2_ (cal mol^−1^)	(2.1 ± 0.2) × 10^3^ c	(−6.3 ± 0.4) × 10^2^ b	(−1.3 ± 0.2) × 10^3^ a
*−T*Δ*S*_2_ (cal mol^−1^)	(−1.51 ± 0.03) × 10^4^ a	(−1.344 ± 0.006) × 10^4^ b	(−1.23 ± 0.02) × 10^4^ c

## Data Availability

The original contributions presented in the study are included in the article, further inquiries can be directed to the corresponding author.

## Supplementary Materials

The following supporting information can be downloaded at: https://www.mdpi.com/article/10.3390/foods13060862/s1, Table S1. Flavan-3-ol composition (%) of the seed extract employed in the study. Table S2. Molecular weight (MW), total amino acid content, and percentages of hydrophobic, polar, acid, basic and aromatic residues of each HMW_SP_ according to the UniProt database [43]. Reference [43] is cited in the Supplementary Materials. Figure S1. Modified Stern–Volmer plot describing tryptophan quenching of HMW_SPs_ by increasing concentrations of seed flavan-3-ols: (a) α-amylase (3 µM); (b) albumin (3 µM). The λ_em_ maximum was recorded at ~350 nm. Table S3. Stern–Volmer quenching constant (*K*_sv_) and bimolecular quenching constant (*K*_q_) for the HMW_SP_/flavan-3-ol interactions.
