# Supplementary material for: Possible Role of High-Molecular-Weight Salivary Proteins in Astringency Development
Source: Foods. 2024 Mar 13;13(6):862. doi: 10.3390/foods13060862 (PMC10969767; doi:10.3390/foods13060862)
Supplement: Supplementary file 1 [file foods-13-00862-s001.zip › foods-2866626-supplementary.pdf]

Table S1. Flavan-3-ol composition (%) of the seed extract employed in the study.

<table>
<tr><td colspan="6">Non-galloylated</td><td rowspan="6">mDP
2.09</td></tr>
<tr><td>Monomers</td><td>Dimers</td><td>Trimers</td><td>Tetrames</td><td>Pentamers</td><td>Total</td></tr>
<tr><td>8.64</td><td>64.66</td><td>13.29</td><td>1.10</td><td>0.29</td><td>87.98</td></tr>
<tr><td colspan="6">Galloylated</td></tr>
<tr><td>Monomers</td><td>Dimers</td><td>Trimers</td><td>Tetrames</td><td>Pentamers</td><td>Total</td></tr>
<tr><td>---</td><td>11.30</td><td>0.55</td><td>0.17</td><td>---</td><td>12.02</td></tr>
</table>

mDP: medium degree of polymerization

Table S2. Molecular weight (MW), total amino acids content and percentages of hydrophobic, polar, acid, basic and aromatic residues of each HMW_{SP} according to UniProt database [43].

HMW_{SP}	MW	Total Amino acids	Hydrophobic residues	Polar residues	Acid residues	Basic residues	Aromatic residues
**α-Amylase** from human saliva	~58kDa	496	39.3%	24%	11.1%	12.7%	12.9%
**Albumin** from human serum	~66 kDa	585	36.6%	21.2%	16.8%	16.9%	8.5%
**Mucin** from bovine submaxillary glands	~58kDa	558	45.7%	37.8%	4.5%	7.3%	4.7%

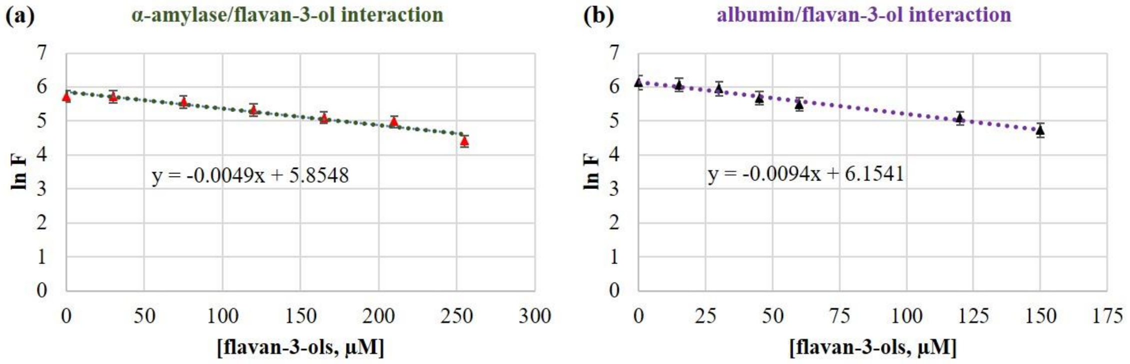

Figure S1. Modified Stern-Volmer plot describing tryptophan quenching of HMW_{SP} by increasing concentrations of seed flavan-3-ols: (**a**) α-amylase (3 µM); (**b**) albumin (3 µM). The λ_{em} maximum was recorded at ~350 nm.

Table S3. Stern-Volmer Quenching Constant (K_{sv}) and Bimolecular Quenching Constant (K_q) for the HMW_{SP}:flavan-3-ol interactions.

HMW_{SP}	τ_0 (s)	K_{sv} (M^{-1})	K_q ($M^{-1} s^{-1}$)
α-amylase	2.97×10^{-9}	4.90×10^{3}*	1.65×10^{12}**
albumin	6.38×10^{-9}	9.40×10^{3}*	1.88×10^{12}**
mucin	3.68×10^{-9}	3.33×10^{4}	9.06×10^{12}

*These values correspond to the Apparent Static Quenching Constant K_{app} (M^{-1})

** These values correspond to the Apparent Bimolecular Quenching Constant K_q^{app} ($M^{-1} s^{-1}$)
